# An EEG-based non-linear state-space model for trust inference in a space-relevant human-autonomy teaming task

**DOI:** 10.3389/fnrgo.2026.1810115

**Published:** 2026-08-03

**Authors:** Yimin Qin, Pinn Prugsanapan, Sarah Leary, Kai Yi Shih, Zhaodan Kong, Allison Hayman, Torin Clark

**Affiliations:** 1 Department of Mechanical and Aerospace Engineering, University of California, Davis, Davis, CA, United States; 2 Department of Computer Science, University of California, Davis, Davis, CA, United States; 3 Department of Aerospace Engineering Sciences, University of Colorado-Boulder, Boulder, CO, United States; 4 Department of Biomedical Engineering, University of California, Davis, Davis, CA, United States

**Keywords:** electroencephalogram (EEG), human-autonomy teaming (HAT), non-linear dynamic system, state-space model, trust inference

## Abstract

Effective collaboration in human-autonomy teaming (HAT) depends on maintaining appropriately calibrated trust in the autonomous partner. Trust evolves as operators observe system behavior and task outcomes, requiring continuous monitoring rather than overall surveys or post-task self-reports. Electroencephalogram (EEG) offers high temporal resolution for tracking cognitive states; however, most existing EEG-based trust models are static or rely on linear mappings that fail to capture the non-linear and time-dependent nature of trust dynamics. This paper presents an EEG-based non-linear state-space model for continuous trust inference during a space-relevant supervisory task. The model integrates a low-dimensional linear latent process to represent the temporal evolution of trust with a non-linear manifold that maps EEG spectral features to the latent state, enabling trust prediction from both latent dynamics and neural representations. Model performance was evaluated using EEG data collected from participants monitoring autonomous systems with varying reliability and transparency, and compared against a linear dynamic model and a static model. After correcting the unit of statistical inference to participant-level averages, the proposed model achieved significantly higher correlation with self-reported trust than both the linear dynamic model (*p* = 0.0021) and the static model (*p* < 0.0001), and significantly lower prediction error than the static model (*p* = 0.0020). The RMSE reduction relative to the linear dynamic model was numerically favorable but not statistically significant after participant-level aggregation. Model interpretation revealed that trust inference relied on distributed neural features, with contributions from frontal and prefrontal regions in delta and alpha bands and additional involvement of higher-frequency activity (low- and mid-gamma bands), and that high- and low-trust states occupy distinct regions of the inferred latent space. These results demonstrate that incorporating non-linear neural structure within a dynamic modeling framework improves EEG-based trust inference and supports the development of cognition-aware adaptive autonomy.

## 1 Introduction

As autonomous systems are increasingly deployed in complex operational environments, effective human-autonomy teaming depends not only on system capability but also on the human operator's trust in the autonomous teammate. Trust influences how humans allocate attention, rely on system outputs, and decide when to intervene, and miscalibrated trust–whether excessive or insufficient–can adversely affect team performance and safety (Parasuraman and Riley, 1997; Lee and See, 2004; Hoff and Bashir, 2015). As a result, understanding how trust develops and changes over time has become an important consideration in the design of trustworthy autonomous systems.

Prior work has shown that trust in automation is neither static nor uniform across individuals. Instead, trust evolves through ongoing interaction, shaped by observed system behavior, performance feedback, and explanatory information provided by the system (Muir and Moray, 1996; Merritt et al., 2013). In human factors research, trust is commonly conceptualized as a dynamic internal state influenced by factors such as system reliability (i.e., the consistency and accuracy with which the system performs its intended task), explainability (i.e., the extent to which the system communicates its state, reasoning, or uncertainty to the human teammate), and prior experience, rather than as a fixed disposition or a post-task evaluation (Lee and See, 2004). These dynamics are especially relevant in co-reliant interaction settings, where humans and autonomous systems must mutually depend on one another to achieve shared task objectives (Endsley and Kiris, 1995; De Visser et al., 2016).

Despite its importance, trust is most often assessed using retrospective questionnaires or aggregated summary measures, which provide limited resolution on how trust evolves during task execution (Schaefer, 2016; Jian et al., 2000). Although continuous or repeated trust reporting can capture temporal variation in trust assessments (Akash et al., 2017; De Visser et al., 2016), self-reported measures alone offer limited insight into the underlying cognitive processes supporting trust formation and adjustment. This limitation has motivated increased interest in psychophysiological and neural sensing approaches for trust inference, with electroencephalogram (EEG) offering a non-invasive means to examine cognitive and affective processes during ongoing human-autonomy interaction (Hopko and Mehta, 2021).

Recent studies have demonstrated the feasibility of inferring trust-related measures from EEG features using machine learning and classification models (Wang et al., 2018; Xu et al., 2024; Zhang et al., 2024); however, many existing approaches treat trust inference as a static regression or classification problem, or rely on models that provide limited interpretability of the learned representations. Such approaches often do not explicitly account for the temporal structure of trust or offer clear insight into which neural features contribute to trust estimation. In operational contexts where adaptive autonomy or transparency mechanisms may depend on interpretable and temporally sensitive trust indicators, these limitations remain a challenge (Hoff and Bashir, 2015).

To address these gaps, the present study investigates EEG-based trust inference using a non-linear dynamic modeling framework that explicitly captures temporal trust evolution while supporting textitpost hoc interpretability. Trust dynamics are examined in an operationally motivated human-autonomy teaming task involving satellite-based surveillance, in which participants interact with autonomous systems that vary in reliability and explainability (Sung et al., 2024). Trust is measured continuously throughout task execution, enabling analysis of both inter-individual variability and within-session trust dynamics.

## 2 Background

### 2.1 Trust evaluation

Trust in automation has long been recognized as a critical factor shaping human interaction with autonomous and decision-support systems. Within human factors and human-autonomy teaming research, trust is generally regarded as a dynamic internal attitude that influences reliance, compliance, and intervention behavior, rather than a fixed trait or discrete decision outcome (Sheridan, 1992; Madhavan and Wiegmann, 2007). Early empirical studies demonstrated that trust evolves through interaction as users observe system performance, interpret feedback, and integrate experience over time, making trust inherently context-dependent and temporally variable (Dzindolet et al., 2003).

The most common approach to trust evaluation relies on subjective self-report instruments administered during or after task execution. These measures assess constructs such as perceived system competence, predictability, and reliability (Jian et al., 2000). While self-report methods are straightforward to deploy and provide interpretable summaries, they typically offer limited temporal resolution and may fail to capture rapid within-task trust fluctuations. In dynamic operational environments, trust ratings collected after the task can still be affected by recall or outcome-related biases, reducing their sensitivity to rapid fluctuations in trust and complicating interpretation of temporal trust trends (Schaefer, 2016).

To better capture temporal variation in trust, several studies have adopted repeated or continuous trust reporting approaches, including interval-based prompts and continuous rating interfaces. These methods have revealed substantial heterogeneity in trust trajectories across individuals, ranging from rapidly fluctuating trust to gradual adaptation or sustained stability over time (Yang et al., 2017; Guo and Yang, 2021). Such findings support theoretical perspectives that characterize trust as an evolving cognitive state updated through ongoing evidence accumulation, rather than a simple monotonic response to system reliability.

Beyond subjective reports, trust has also been inferred indirectly through behavioral indicators such as reliance frequency, automation usage patterns, monitoring behavior, and intervention timing (Boubin et al., 2017; Körber et al., 2018; Kohn et al., 2021). Although these behavioral measures provide valuable insight into trust-related action, they are inherently task-specific and may reflect workload, strategy, or situational constraints in addition to trust itself, as reliance decisions are influenced not just by latent trust but also by context and system characteristics adjustment (Parasuraman and Riley, 1997; Lee and See, 2004).

The limitations of subjective and behavioral approaches have motivated interest in physiological and neurophysiological methods for trust evaluation that aim to infer trust from signals associated with cognitive and affective processing, enabling more continuous and unobtrusive assessment (Ajenaghughrure et al., 2020; Kohn et al., 2021). In particular, EEG has been explored due to its sensitivity to attentional engagement, cognitive control, and decision-related neural activity and its feasibility for real-time trust monitoring (Zhang et al., 2024). Neural measures including EEG have been proposed as alternative trust indicators because traditional questionnaires cannot readily support adaptive, real-time systems (Hopko and Mehta, 2021). However, many EEG-based trust studies continue to treat trust as a static outcome or rely on predictive models with limited interpretability. Additionally, most employ static statistical methods rather than dynamic analysis. This restricts insight into how trust evolves over time or how neural features contribute to trust inference (Ajenaghughrure et al., 2020).

Given these premises, prior findings highlight the need for trust evaluation methods that can capture temporal variation and individual differences with minimal disruption to task performance, while maintaining interpretability and theoretical grounding. These requirements motivate the use of EEG-based dynamic modeling approaches that represent trust as a latent internal state evolving over time.

### 2.2 EEG-based trust inference

Recent advances in physiological sensing have increased interest in inferring trust from neural activity, with EEG emerging as a practical and informative modality (Ajenaghughrure et al., 2020; Kohn et al., 2021; Chen et al., 2025). EEG offers non-invasive measurement with millisecond-level temporal resolution, making it sensitive to cognitive and affective processes relevant to trust (e.g., attention allocation, workload, uncertainty/outcome evaluation, and performance/decision monitoring) (Klimesch, 2012; Gevins et al., 1998; Schaefer A. et al., 2016; Cavanagh and Frank, 2014; Holroyd and Coles, 2002). These characteristics are well-aligned with human-autonomy teaming, where trust can adjust quickly as operators observe system behavior and outcomes (Lee and See, 2004; Hoff and Bashir, 2015).

Early EEG studies related to trust and automation primarily examined associations between task conditions and spectral activity within specific frequency bands or scalp regions, often relying on condition-level or group-level comparisons rather than individualized temporal modeling (Hu et al., 2016; Akash et al., 2018). Prior work has linked frontal theta and alpha activity to cognitive control and mental workload (Klimesch, 1999; Cavanagh and Frank, 2014), while beta and gamma activity have been associated with decision confidence, evidence integration, and higher-order cognitive processing (Makeig et al., 2004; Wyart et al., 2012; Pisauro et al., 2017; De Vico Fallani et al., 2010). Collectively, these findings suggest that trust-related neural activity is distributed across multiple frequencies and cortical regions rather than localized to a single neural source. However, much of this work relied on static or aggregated analyses at the condition or group level, which limits insight into individual variability and the within-task evolution of trust over time (Akash et al., 2018).

More recent studies have moved toward predictive modeling approaches that map EEG features directly to trust measures. A range of machine learning techniques, such as ensemble methods, support vector machines, and deep neural networks, have been applied to classify or regress trust states from EEG-derived features (Akash et al., 2018; Zhang et al., 2024; Jui et al., 2025). These efforts demonstrate the feasibility of neural trust inference, but typically frame trust as a static outcome or assume independence across time points, with models trained on aggregated feature windows or isolated samples, limiting their ability to capture temporal dependencies intrinsic to trust formation and adaptation, particularly in extended or multi-stage interactions with autonomous systems (Zhang et al., 2024; Jui et al., 2025). In addition, model interpretability presents an additional challenge in EEG-based trust inference. Although more complex models can improve prediction accuracy, they often obscure the contribution of individual neural features (Doshi-Velez and Kim, 2017; Lundberg and Lee, 2017). In the context of HAT, such opacity limits confidence in trust estimates and complicates validation, transparency, and ethical deployment (Gunning and Aha, 2019). This has motivated increasing interest in combining predictive models with *post hoc* interpretability methods, such as local feature-attribution techniques, that can clarify how specific spectral and spatial EEG features contribute to trust estimation (Ribeiro et al., 2016; Lundberg and Lee, 2017).

Despite these advances, several gaps remain. Few EEG-based studies explicitly represent trust as a latent dynamic process, despite strong theoretical and empirical evidence that trust evolves over time (Lee and See, 2004; Hoff and Bashir, 2015; Schaefer K. E. et al., 2016). Moreover, limited attention has been given to how neural representations of trust differ across individuals or across distinct trust dynamics (Sheridan, 2019; Chung and Yang, 2024). Finally, the relationship between temporal trust patterns and their neural correlates remains insufficiently characterized, and current psychophysiological trust inference approaches still face application-specific validation and generalizability limitations (Ajenaghughrure et al., 2020; Rodriguez Rodriguez et al., 2023).

Addressing these challenges requires modeling frameworks that integrate temporal dynamics, non-linear neural representations, and interpretable feature attribution. Such approaches enable continuous trust inference and provide a principled means to examine how trust emerges, adapts, and stabilizes in neural activity during ongoing interaction with autonomous systems.

### 2.3 Research objectives and contributions

Although trust in HAT is widely recognized as a dynamic construct, most existing evaluation and inference approaches remain static or treat trust as a sequence of independent observations. Prior EEG-based studies have demonstrated feasibility but often lack explicit temporal modeling and offer limited interpretability of the neural processes underlying trust, constraining their utility for adaptive autonomy.

The objective of this work is to develop and evaluate an EEG-based trust inference framework that models trust as a latent, time-evolving internal state while maintaining interpretability of neural contributions. Drawing inspiration from a dynamical flexible inference for non-linear embeddings framework (Abbaspourazad et al., 2024), we propose a non-linear dynamic model that integrates learned neural manifolds with state-space dynamics to capture both the structure and temporal evolution of trust. The pipeline is evaluated in an operationally motivated HAT task and compared against linear dynamic and static baseline models. Beyond predictive performance, *post hoc* explanation is applied to the proposed model to examine how spectral EEG features across channels and frequency bands contribute to trust inference. By jointly analyzing trust distributions, representative temporal dynamics, and feature-importance patterns, the approach accounts for individual variability and links neural signatures to distinct trust behaviors.

Overall, this work aims to advance EEG-based trust inference by demonstrating that incorporating non-linear structure and temporal dynamics improves both predictive accuracy and interpretability. The findings inform the design of trust-aware autonomous systems capable of monitoring and responding to evolving human trust states in real time.

## 3 Methodology

### 3.1 Experimental design

#### 3.1.1 Task overview

To examine the temporal dynamics of trust in HAT, this work recruited subjects to perform an operationally motivated decision-support task in collaboration with a simulated autonomous system. The task was designed to emulate satellite-based surveillance operations, in which the main goal was to identify ground troop movement with the assistance of autonomous systems. A detailed description of the task paradigm has been reported previously by our research team (Sung et al., 2024), and a summary relevant to the present study is provided here.

**Figure 1:**
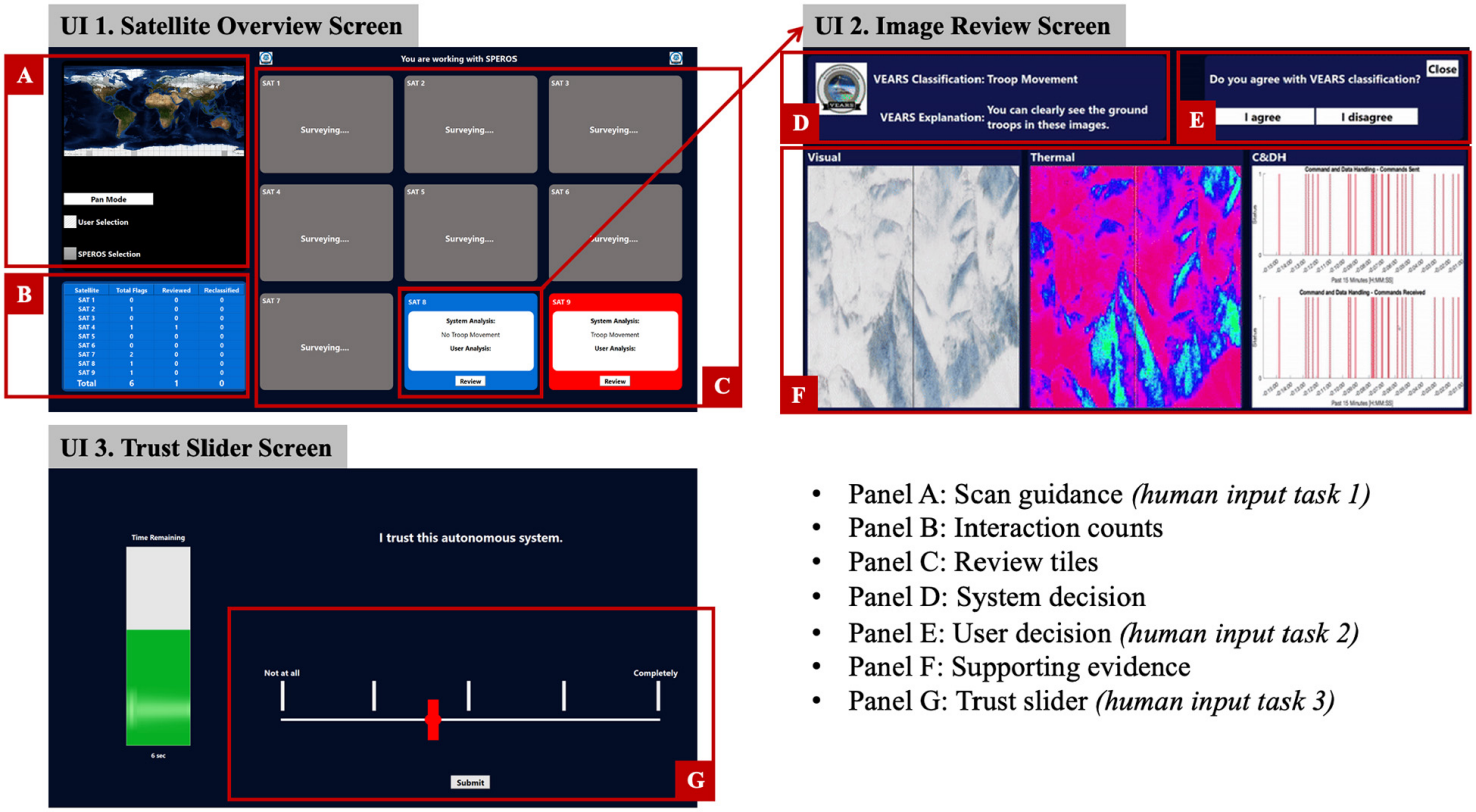
Task interfaces and trust reporting screens used in the experiment. The interfaces include Panels **(A–C)** a satellite overview and task management screen, **(D–F)** an image review and decision-making screen displaying system outputs and supporting evidence from the satellite system, and **(G)** a trust slider screen for continuous self-reported trust input every 30 s.

During the experiment, subjects interacted with an autonomous system through a graphical user interface displayed on a computer monitor (Figure 1). The primary interface (UI 1 - Satellite Overview Screen) presented information from nine simulated satellites review boxes. For each box, the autonomous system generated a binary classification indicating whether troop movement was detected. These classifications were displayed as individual review tiles (Panel C), allowing participants to monitor the system's assessments across satellites in parallel. Specifically, only two boxes were reviewable each time, and Panel C was refreshed every 30 s. Additionally, to support co-reliant interaction between human and system, subjects were also tasked with assisting the autonomous system in guiding satellite image acquisition. This scan guidance task (Panel A) required participants to interact with a global map and suggest geographic regions for satellite imaging. A summary panel (Panel B) provided cumulative interaction counts, including the number of system flags reviewed and reclassified by the participant. When a participant elected to review a system classification, the interface transitioned to the Image Review Screen (UI 2). This screen displayed the autonomous system's decision and associated explanation (Panel D), along with the participant's response options (Panel E). Supporting evidence was presented in the form of multi-modal satellite data, including visual map, thermal map, and command and data handling plot timelines (Panel F). Participants could either agree or disagree with the system's classification, thereby directly influencing task outcomes.

Trust was explicitly measured using a dedicated Trust Slider Screen (UI 3), presented at regular intervals (30 s) during the task. Participants reported their momentary trust in the autonomous system using a continuous slider ranging from “not at all” to “completely” (Panel G). Trust ratings were sampled repeatedly throughout each session, yielding a continuous time series that captured within-session trust evolution rather than a single post-task assessment.

What's more, an incentive mechanism was embedded in the task design, where subject compensation was tied to both the quantity and accuracy of reclassification decisions. That said, participants could either increase or decrease their final compensation based on performance. This structure introduced tangible risk and reward, providing meaningful stakes and sufficient motivation to engage with the task. Together, these design elements created a task environment in which trust could evolve dynamically as participants balanced system monitoring, evidence review, and scan guidance under varying levels of system reliability and explainability.

#### 3.1.2 Experiment procedure

**Figure 2:**
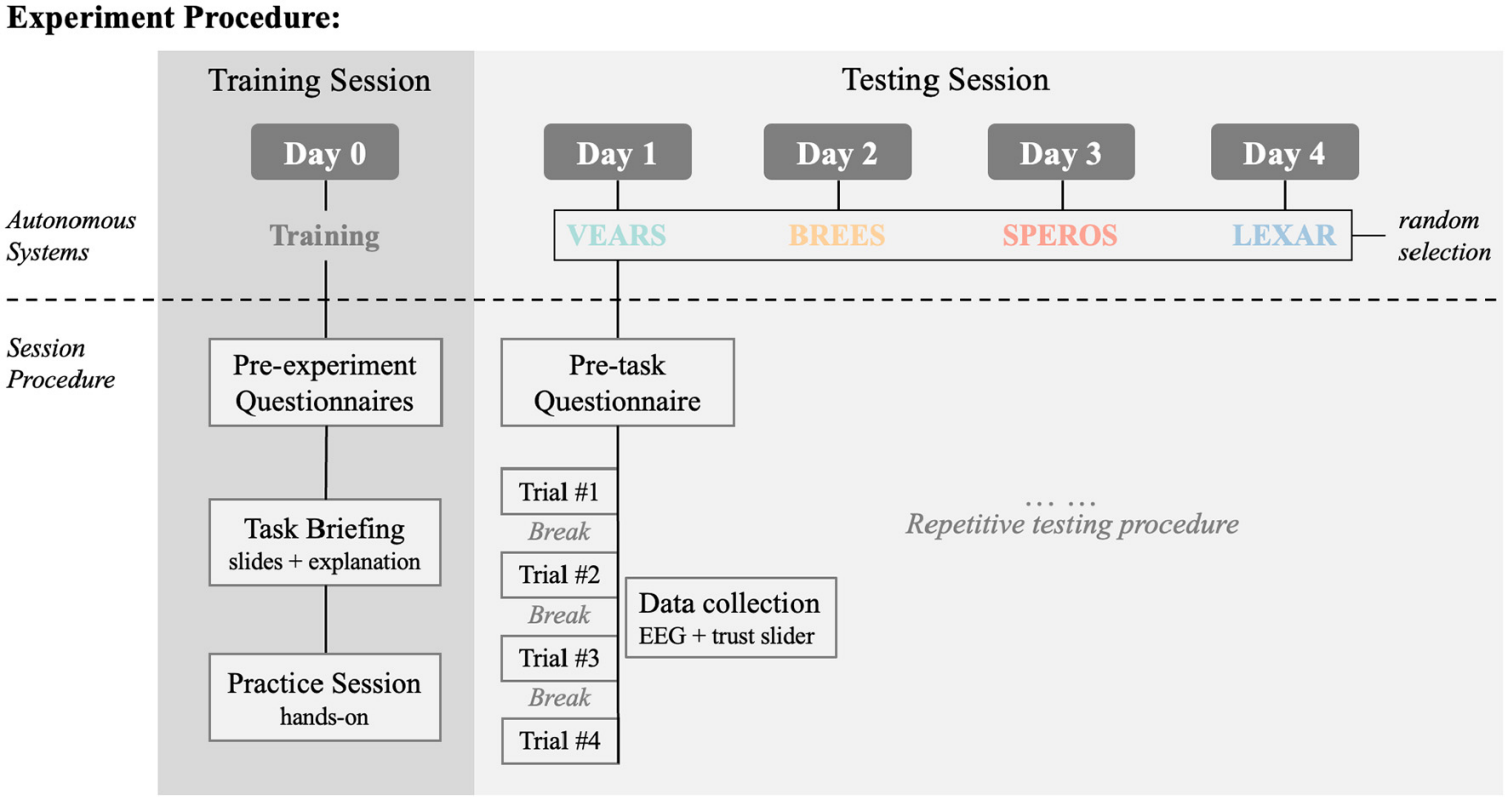
Experimental procedure and session timeline. The study consisted of a training session (Day 0) followed by four testing sessions (Days 1–4).

The experiment was conducted over five sessions, consisting of an initial training session followed by four testing sessions on separate days (Figure 2). The training session took place on Day 0 and was designed to familiarize subjects with the task objectives, interface layout, and interaction procedures before data collection began. At the start of the training session, subjects completed a set of pre-experiment questionnaires that collected demographic information as well as baseline measures related to prior experience and individual dispositions. Subjects then received a structured task briefing, during which the experimenter presented standardized instructional slides and explained the task workflow, interface elements, and decision-making requirements in detail. This briefing emphasized the collaborative nature of the task and clarified the participant's role in monitoring the autonomous system, reviewing system decisions, providing scan guidance, and reporting trust. To reduce variability in how participants interpreted the trust slider, all participants were given the same task-specific definition of trust during training. Specifically, participants were instructed to report trust as “your attitude that the autonomous system will help you achieve your goals given the uncertainty and vulnerability associated with this task (and this task only).” They were further instructed not to consider how the system might perform in other tasks, and to base their slider response on both perceived system performance and their feelings toward the autonomous system. This standardized wording was presented before experimental data collection and was used to anchor the repeated trust reports collected during the testing sessions. Following the briefing, subjects completed a hands-on practice session using a training version of the autonomous system. This practice phase allowed them to gain experience with the interface, review process, and trust reporting mechanism without any experimental data being recorded, and none of the experimental system conditions were used during training.

After the training session, subjects completed four testing sessions on separate days (Days 1–4). During each testing session, they interacted with one autonomous system variant, of which assignment was randomized across days. Subjects were told that they would be working with a different autonomous system in each session but were not informed of the specific characteristics of the system. To effectively vary trust-related factors, the autonomous system was designed at varied reliability and explainability levels. System reliability referred to the accuracy of the system's troop movement classification. A low-reliability condition provided correct classifications approximately two-thirds of the time and a high-reliability condition with substantially higher accuracy. In addition, the system's explanatory style was manipulated to alter affective components of trust: in low-explainability conditions, explanations were terse and machine-like, whereas in high-explainability conditions, they employed more naturalistic language. Reliability and explainability were crossed to create four distinct autonomous system variants: VEARS (high reliability, high explainability), BREES (low reliability, high explainability), SPEROS (high reliability, low explainability), and LEXAR (low reliability, low explainability).

At the beginning of each testing session, subjects completed a brief pre-task questionnaire to capture session-specific baseline measures, such as sleeping hours and alcohol consumption last night. Subjects then completed four task trials within the session, with short breaks provided between trials to reduce fatigue. Based on recorded task logs, the active task portion of each trial lasted approximately 10.9 min (mean = 652 s, SD = 10.5 s; median = 650 s; range = 636–688 s), preceded by a short baseline period (median = 35 s; mean = 45 s; range = 35–131 s). The resulting total trial duration was approximately 11.6 min on average. Short participant-paced breaks were provided between trials; recorded same-session break intervals had a median duration of approximately 3.0 min (IQR = 2.6–4.1 min), with longer breaks allowed when requested. During each trial, participants performed the satellite monitoring task described previously, including providing scan guidance, optionally reviewing system classifications, and interacting with supporting evidence presented by the autonomous system. EEG data and continuous trust ratings were collected throughout task execution. Trust was reported every 30 s using the trust slider interface, yielding a continuous trust signal for each trial. Apart from the autonomous system variant, the testing procedure remained identical across all sessions, enabling within-subject comparison of trust dynamics and neural responses under different combinations of system reliability and explainability.

### 3.2 Data analysis

**Figure 3:**
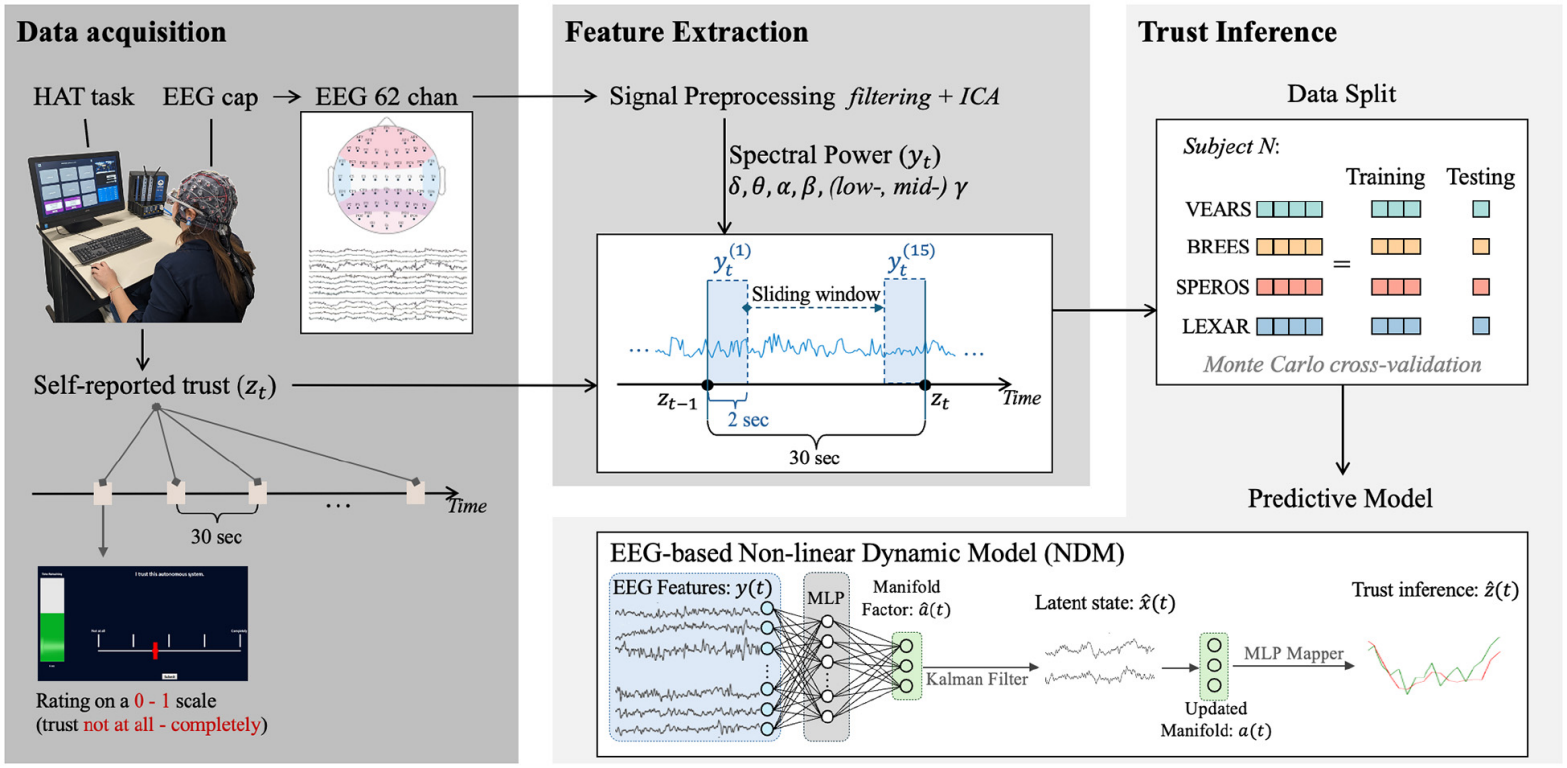
Overview of the EEG-based trust inference pipeline. EEG signals were recorded during a human-autonomy teaming task and preprocessed using filtering and ICA, followed by spectral power extraction across multiple frequency bands. EEG features were aligned with self-reported trust ratings using a sliding-window scheme. Model training and testing were performed using Monte Carlo cross-validation, and trust was inferred using a non-linear dynamic model.

This section describes the data analysis pipeline used to process neural and trust data and to infer trust dynamics from EEG signals. An overview of the analysis workflow is illustrated in Figure 3, which summarizes the progression from data acquisition, feature extraction and model generation. The following subsections detail each stage of the pipeline, including the collection and synchronization of EEG and trust data, the extraction of spectral EEG features, and the development of trust inference models used for subsequent performance evaluation and interpretation.

#### 3.2.1 Data acquisition

Data used in this study were collected from ten subjects at the University of California, Davis under an approved Institutional Review Board (IRB) protocol (Protocol No. 2033250-3). Participants were recruited from the university community and met the study inclusion criteria, including adult age, normal or corrected-to-normal vision, and ability to complete the computer-based task instructions in English. Formal education level was not analyzed as a study variable in the present modeling work. However, the pre-experiment questionnaire assessed demographic variables and task-relevant prior experience, including prior use of robotic or autonomous systems, navigation aids, video games, aerospace or spaceflight displays, and military or ground-troop monitoring displays. These questionnaire items were added here to clarify the sample characteristics and the population to which the present findings most directly generalize. EEG data were collected concurrently with task-related trust evaluations and reclassification decisions while participants performed the HAT task described previously. Neural activity was recorded using a 62-channel g.tec active-electrode headset with a sampling rate of 500 Hz. EEG signals were continuously acquired throughout task execution, including periods of system monitoring, evidence review, scan guidance, and trust reporting.

In parallel with EEG recording, participants provided self-reported trust ratings using a continuous slider interface. Trust was rated on a normalized 0–1 scale, anchored from “not at all” to “completely.” Trust ratings were sampled at regular intervals during each trial, yielding a time-resolved self-reported trust criterion *z*_*t*_ that captured the evolution of trust across task execution. To ensure precise temporal alignment across data streams, the Lab Streaming Layer (LSL) framework was employed. EEG samples and trust-report events were streamed through LSL and time-stamped using a shared system clock with sub-millisecond accuracy.

#### 3.2.2 Feature extraction

Raw EEG signals were first preprocessed using standard procedures, including bandpass filtering on 0.5–75 Hz, notch filtering at 60 Hz, and artifact removal via independent component analysis (ICA). The 0.5–75 Hz bandpass and 60 Hz notch filtering were selected to attenuate slow drift, high-frequency noise, and line noise while preserving the spectral range used in subsequent feature extraction (Delorme and Makeig, 2004; Cohen, 2014). Following preprocessing, spectral power features were extracted from each EEG channel.

EEG spectral power was computed within six frequency bands: delta (δ, 1–4 Hz), theta (θ, 4–8 Hz), alpha (α, 8–13 Hz), beta (β, 13–30 Hz), low-gamma (low-γ, 30-50 Hz), and mid-gamma (mid-γ 50–70 Hz). These ranges follow commonly used EEG spectral definitions and prior cognitive/neuroergonomic studies linking low-frequency and gamma-band activity to attention, cognitive control, workload, memory, and information integration (Klimesch, 1999; Cavanagh and Frank, 2014; Borghini et al., 2014; Jensen et al., 2007; Fries, 2015). Feature extraction was performed using a sliding-window approach, where spectral power was computed with the Power Spectral Density of the EEG signal over 2-s windows and aggregated to correspond with the temporal resolution of trust reporting (Figure 3). Specifically, EEG features preceding each trust rating were summarized to form the observation vector **y**_*t*_, representing neural activity associated with the trust state at time t.

This process resulted in a high-dimensional EEG feature vector at each trust update, comprising spectral power values across all 62 channels and six frequency bands.

#### 3.2.3 Model generation

To evaluate the proposed EEG-based non-linear dynamic model, two baseline models were implemented for comparison: a linear dynamic model and a linear static model. All models aim to infer continuous trust values ẑ_*t*_∈[0, 1] from high-dimensional EEG features *y*_*t*_, but differ in how temporal structure and feature-trust relationships are modeled. The mathematical formulations of the proposed and baseline models are provided in Equations 1–11.

i) Non-linear dynamic model (NDM)

The proposed model treats human trust as a latent dynamic process that evolves over time and is indirectly reflected in EEG features. The model structure builds on the dynamical flexible inference for non-linear embeddings framework (Abbaspourazad et al., 2024), which combines non-linear embedding functions with a linear state-space model to support filtering and smoothing in a low-dimensional manifold. In the present work, this framework was adapted for EEG-based trust inference by using EEG spectral power features as high-dimensional observations and by adding a supervised mapper from the inferred manifold state to self-reported trust. The latent dynamic component follows standard state-space modeling and Kalman filtering/smoothing principles (Kalman, 1960; Rauch et al., 1965).

Let **y**_*t*_ denote the EEG spectral feature vector at time *t*, ${\tilde{\text{a}}}_{t}$ the encoded manifold observation, **x**_*t*_ the dynamic latent state, and *z*_*t*_ the self-reported trust criterion. The NDM first encodes the EEG feature vector into a low-dimensional manifold observation:


$$\begin{array}{l} {\tilde{\text{a}}}_{t}=g_{\theta }\left({\text{y}}_{t}\right), \end{array}$$ (1)

where *g*_θ_(·) is a non-linear encoder implemented as a multilayer perceptron (MLP). The encoded manifold observations are then modeled with a linear state-space process:


$$\begin{array}{l} {\text{x}}_{t+1}=A{\text{x}}_{t}+{\text{w}}_{t},\;{\text{w}}_{t}\sim N\left(0,\text{W}\right), \end{array}$$ (2)

$$\begin{array}{l} {\tilde{\text{a}}}_{t}=C{\text{x}}_{t}+{\text{r}}_{t},\;{\text{r}}_{t}\sim N\left(0,\text{R}\right). \end{array}$$ (3)

Here, A defines the temporal transition of the dynamic latent state and C maps the dynamic latent state x_*t*_ to the manifold observation ${\tilde{\text{a}}}_{t}$. Thus, C is not a direct trust-observation matrix; trust is predicted by a separate supervised non-linear mapper. After Kalman filtering and Rauch–Tung–Striebel smoothing, the filtered and smoothed manifold estimates are obtained as ${\hat{\text{a}}}_{t|t}=\text{C}{\hat{\text{x}}}_{t|t}$ and ${\hat{\text{a}}}_{t|T}=\text{C}{\hat{\text{x}}}_{t|T}$. EEG reconstruction and trust prediction are then given by:


$$\begin{array}{l} {\hat{\text{y}}}_{t}=f_{\phi }\left({\hat{\text{a}}}_{t}\right), \end{array}$$ (4)

$$\begin{array}{l} {ẑ}_{t}=h_{\psi }\left({\hat{\text{a}}}_{t|T}\right), \end{array}$$ (5)

where *f*_ϕ_(·) is the non-linear decoder and *h*_ψ_(·) is the supervised trust mapper. The reported offline evaluation used smoothed states; in an online adaptive-autonomy setting, the same framework could use causal filtered states ${\hat{\text{x}}}_{t|t}$.

The NDM was trained using a composite objective that jointly encouraged multi-step EEG prediction/reconstruction and trust prediction:


$$\begin{array}{l} L\left(\phi ,\theta ,\psi \right)=\underset{k\in K}{\sum }\overset{T-k}{\underset{t=1}{\sum }}\ell_{y}\left({\text{y}}_{t+k},{\hat{\text{y}}}_{t+k|t}\right)+{\text{λ}}_{trust}\overset{T}{\underset{t=1}{\sum }}M_{t}\ell_{z}\left(h_{\psi }\left({\hat{\text{a}}}_{t|T}\right),z_{t}\right)\\ \;+{\text{λ}}_{reg}L_{reg}. \end{array}$$ (6)

Here, ℓ_*y*_(·) denotes EEG prediction/reconstruction error, ℓ_*z*_(·) denotes trust prediction error, K = {1, 2} denotes the prediction horizons used in this study, and *M*_*t*_ is a binary mask indicating time points with available self-reported trust ratings. This formulation clarifies that the encoder, state-space model, decoder, and supervised trust mapper are trained jointly.

ii). Linear dynamic model (LDM)

The linear dynamic model removes the non-linear manifold mapping while retaining the temporal state-space structure. In this model, EEG features are assumed to be linearly related to the latent dynamic state, which is defined as:


$$\begin{array}{l} x_{t+1}=Ax_{t}+\omega \end{array}$$ (7)

$$\begin{array}{l} y_{t}=C_{y}x_{t}+\nu \end{array}$$ (8)

$$\begin{array}{l} z_{t}=C_{z}x_{t}+\epsilon \end{array}$$ (9)

where *x*_*t*_ represents the latent trust-related state, and *y*_*t*_ and *z*_*t*_ denote EEG features and trust, respectively. All mappings are linear, and state estimation is performed using a Kalman filter. Compared to the NDM, this model captures temporal evolution but assumes a linear relationship between EEG features, latent state, and trust. It therefore serves as a baseline to evaluate the contribution of non-linear manifold learning.

iii Linear static model (LSM)

The linear static model ignores temporal dynamics entirely and treats trust inference as an instantaneous regression problem. Each trust value is predicted independently from EEG features at the same time step. The model is expressed as:


$$\begin{array}{l} ẑ=\beta_{0}+y^{⊤}\beta \end{array}$$ (10)

where β denotes regression coefficient. To handle the high dimension challenge of EEG features, LASSO regularization is applied:


$$\beta^{LASSO}=\underset{\beta \in {\mathbb{R}}^{N}}{argmin}\left\{\frac{1}{N}{\left\|z-Y^{⊤}\beta \right\|}_{2}^{2}+\text{λ}{\left\|\beta \right\|}_{1}\right\}$$ (11)

Comparing with the other two dynamic models, it serves as a non-dynamic baseline, isolating the effect of temporal modeling by excluding any state evolution or history dependence.

Model training and evaluation were conducted using a Monte Carlo cross-validation strategy to ensure balanced and robust performance assessment, which was applied uniformly to all models. For each subject, trials were grouped by autonomous system condition. In each iteration, one trial from each condition was randomly selected to form the testing set, while the remaining trials were used for training. This random train-test partitioning was repeated twenty times, and performance metrics were averaged across iterations. Identical data splits were applied to all three models to enable fair comparison. For group-level statistical inference, cross-validation iterations were not treated as independent samples. Instead, performance was first averaged across iterations within each subject, and all paired statistical tests were conducted on the ten independent subject-level averages. Table 1 summarizes the implementation details and hyperparameter settings for the nonlinear dynamic model.

**Table 1:** Implementation details and hyperparameter settings for the non-linear dynamic model.

Component	Setting used in this study
EEG input dimension	372 spectral features for channel-level six-band EEG features (62 channels × 6 bands)
Dynamic latent dimension	dim(x_*t*_) = 2
Manifold latent dimension	dim(a_*t*_) = 2
Encoder/decoder MLP	Fully connected MLPs with tanh activation; hidden layers selected from [16,16,16,16] or [20,20,20,20]
Trust mapper MLP	Fully connected MLP with tanh activation and hidden layers [20,20,20,20]
Prediction horizons	*k* = 1 and *k* = 2 steps ahead
Trust-supervision weight	Grid over 50, 100, 150, and 200
Learning-rate schedule	Cyclic triangular scheduler
Base learning rate	Grid over 0.0001, 0.0005, 0.001, and 0.005
Maximum learning rate	Grid over 0.01 and 0.02
Cycle step size	Grid over 15, 20, and 25
Batch size	12
Maximum training epochs	500
Early stopping	Patience of 20 epochs in the training workflow
Optimizer	Adam with ϵ = 10^−8^
State-space noise	Diagonal W and R, trainable through log-diagonal parameterization
Initial scales	A identity scale = 1; C random scale = 1; W and R initial scale = 0.5; initial covariance = 1

Several design choices were used to reduce overfitting risk in light of the modest participant sample. First, the dynamic and manifold latent dimensions were fixed at two, forcing the model to explain high-dimensional EEG features through a compact state representation. Second, the encoder, decoder, and trust mapper were small fully connected networks selected from bounded hyperparameter grids rather than large unconstrained architectures. Third, all baseline and non-linear models were evaluated using the same subject-specific cross-validation splits, and final statistical inference was performed only on subject-level averages. Finally, model performance was interpreted conservatively, with generalization claims limited to the present intensive repeated-measures EEG design.

#### 3.2.4 Model interpretation

To interpret how neural features contributed to trust inference, a *post hoc* model interpretation analysis was conducted for the proposed NDM. Interpretation was not applied to the baseline models, as they were included primarily for performance comparison in this study.

Local Interpretable Model-agnostic Explanations (LIME) (Ribeiro et al., 2016) was employed to estimate the contribution of individual EEG features to the NDM's trust predictions. LIME approximates the model's behavior locally around a given prediction using a sparse linear surrogate model, enabling attribution of importance scores to input features without requiring access to internal model gradients. This approach is particularly suitable for complex non-linear architectures such as the NDM. For each prediction instance, LIME generated importance values for all EEG spectral features, reflecting their relative influence on the predicted trust output. In this study, feature importance values were computed across test samples and aggregated across Monte Carlo cross-validation iterations. They were further averaged across subjects to facilitate group-level analysis and visualization. It was intended to provide insight into the neural features leveraged by the NDM during trust inference, rather than to establish causal neural mechanisms.

While LIME provides insight into which neural features contribute to trust inference, it does not directly reveal how trust-related information is organized within the learned latent state. To further examine the structure of the inferred latent dynamics, we employed supervised Uniform Manifold Approximation and Projection (UMAP) to visualize the geometry of the learned latent state space. It is a non-linear dimensionality reduction technique that incorporates label information during embedding, encouraging separation of samples associated with different outcome classes while preserving local neighborhood structure in the original space. In this study, supervised UMAP was applied to the inferred smoothed latent state *x*_smooth_. Moreover, trust labels were defined using subject-specific percentile thresholds, with the top and bottom 30% of trust values corresponding to high- and low-trust states, respectively. This percentile-based approach emphasizes relative trust states within individuals instead of using absolute trust scale usage across subjects. By visualizing the latent state trajectories of different trust states, this analysis aims to assess whether high- and low-trust states occupy distinct regions of the learned latent space and whether such structure differs across representative trust dynamics. Importantly, this visualization is used for qualitative analysis and interpretation rather than quantitative model evaluation.

## 4 Results

### 4.1 Trust data characteristics

**Figure 4:**
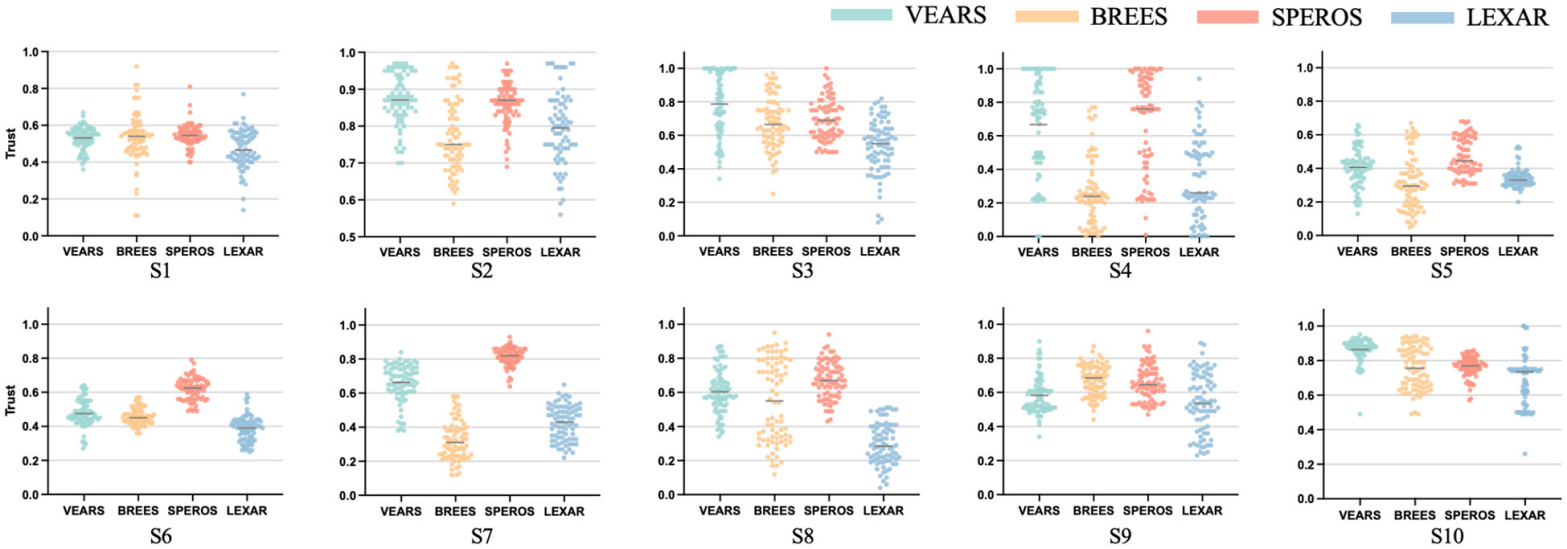
Distribution of self-reported trust ratings across subjects and autonomous systems. Each subplot shows trust ratings collected during interactions with different systems for an individual subject (S1–S10). The distributions illustrate substantial inter-subject and inter-system variability in perceived trust.

Figure 4 illustrates the distribution of self-reported trust ratings across all subjects under each autonomous system condition. Trust was reported on a continuous scale from 0 to 1 throughout the task and is visualized here by subject and system to characterize baseline trust behavior prior to model-based inference. Based on the results, trust ratings exhibited individual variability in both central tendency and dispersion. Some subjects maintained relatively stable trust levels across systems, whereas others showed evident shifts in trust depending on the autonomous system encountered. This variability highlights the personalized nature of trust formation and supports the need for subject-specific modeling approaches.

In addition to individual variability, system-dependent differences in trust distributions were also figured. In general, systems associated with higher reliability and/or higher explainability tended to elicit higher median trust values, while lower reliability-explainability configurations showed reduced trust levels and greater dispersion. However, these trends were not uniform across subjects, and several individuals displayed overlapping trust distributions across systems. Within-subject distributions further revealed distinct trust profiles, including tightly clustered ratings representing stable trust levels over time, as well as broader distributions reflecting fluctuating or context-sensitive trust judgments. These heterogeneous patterns motivated the subsequent analysis of trust dynamics and provided the empirical basis for categorizing representative trust behaviors examined later in this section (Section 4.2).

In brief, the observed trust distributions demonstrate that trust during human-autonomy teaming varied across users or system designs, underscoring the importance of modeling both temporal dynamics and individual differences in trust inference.

### 4.2 Trust temporal dynamics

**Figure 5:**
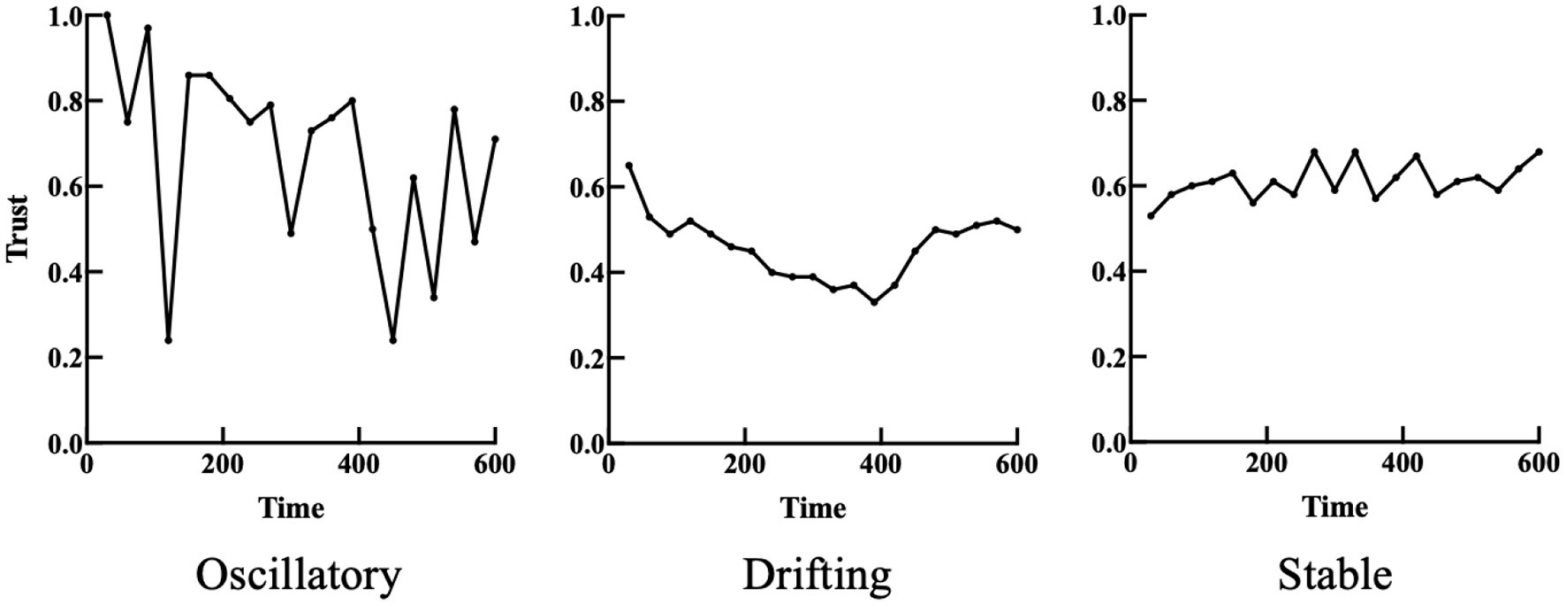
Representative examples of the three identified trust dynamics. Oscillatory dynamics (from S4) exhibit rapid fluctuations between high and low trust values, drifting dynamics (from S7) show gradual trends over time, and stable dynamics (from S5) remain bounded within a narrow range. The three profiles are descriptive representative examples rather than formal labels used for model training or statistical inference.

Motivated by the heterogeneous trust patterns described above, we characterize three representative types of trust temporal dynamics observed across participants. Figure 5 presents three representative temporal patterns of trust observed across subjects during the task. Although trust was reported on a discrete time scale (every 30 s), the trajectories reveal distinct dynamic behaviors during the interaction with the autonomous system. These profiles were used as descriptive representative examples rather than as formal labels for model training or inferential statistics. Representative trials were selected using simple trajectory descriptors: oscillatory dynamics were characterized by high amplitude and high total variation, stable dynamics by low range and low standard deviation, and drifting dynamics by intermediate variability with a sustained low-frequency change over the trial. For the examples shown in Figure 5, the oscillatory trajectory had the largest variability (range = 0.76, SD = 0.223, total variation = 4.79), the stable trajectory remained within a narrow trust band (range = 0.15, SD = 0.042), and the drifting trajectory showed intermediate variability (range = 0.32, SD = 0.077, total variation = 0.63). These patterns were consistently observed across multiple subjects and system conditions and are used here to characterize typical trust dynamics.

- The *Oscillatory* pattern is characterized by frequent and evident fluctuations between relatively high and low trust values. Trust in these cases appears highly sensitive to recent system behavior or contextual cues, resulting in rapid oscillations rather than gradual adaptation. Such trajectories suggest a reactive trust process in which confidence in the autonomous system is repeatedly reassessed rather than accumulated over time.
- The *Drifting* pattern shows a gradual change in trust, either decreasing or increasing during a trial, often with mild short-term variability on a longer-term trend. This behavior reflects a cumulative trust adjustment process, where trust evolves progressively as evidence is integrated over repeated interactions. Compared to the *Oscillatory* pattern, trust updates in these cases are smoother and less influenced by isolated events.
- The *Stable* pattern shows trust remaining within a narrow range throughout the task, with only minor fluctuations around a relatively constant level. These trajectories indicate a conserved trust state, where participants maintain a consistent perception of the autonomous system despite ongoing interaction. Such behavior may reflect strong prior expectations or limited sensitivity to short-term system performance changes.

Overall, these three patterns highlight the heterogeneity of trust dynamics across individuals. Rather than following one single typical trajectory, trust during human-autonomy teaming can manifest as reactive, adaptive, or conserved over time. These observations bring the need for trust predictive models that can accommodate diverse temporal behaviors.

### 4.3 Model performance

**Figure 6:**
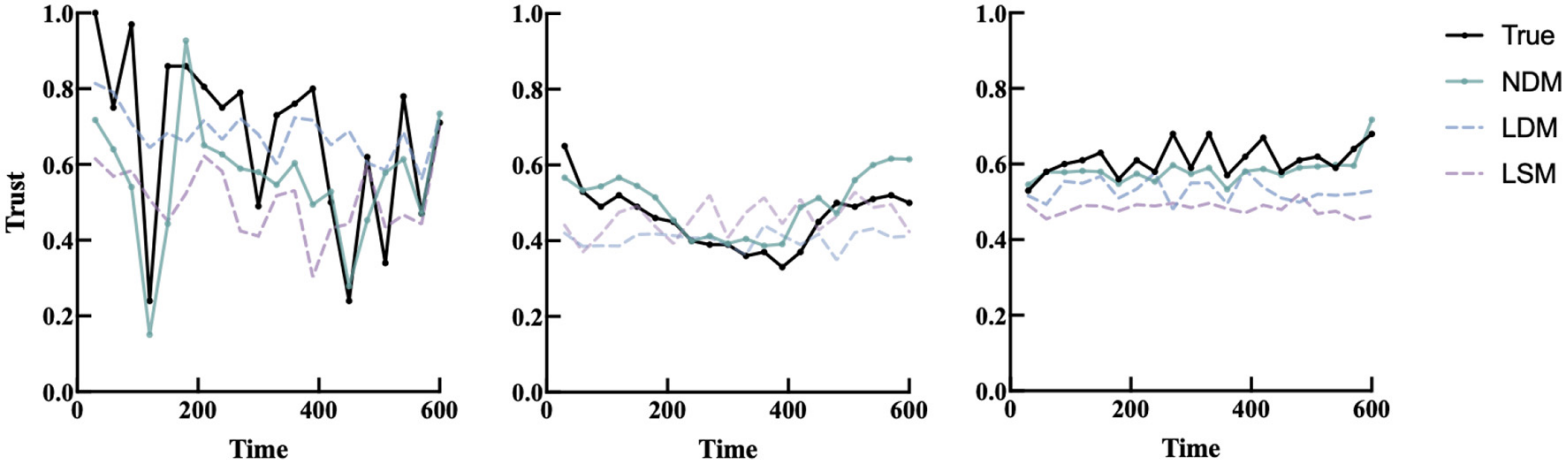
Example trust prediction trajectories for representative trials. Self-reported trust ratings are compared with predictions generated by NDM, LDM, and LSM. The three panels correspond to oscillatory, drifting, and stable trust dynamics, respectively.

To examine how different modeling approaches capture these diverse trust temporal dynamics, we next compare the predictive performance of three trust inference models. Figure 6 illustrates the three representative trust trajectories discussed in the previous section. For each case, predicted trust trajectories from all three models are shown alongside the self-reported trust criterion. For the Oscillatory case, the NDM closely followed rapid fluctuations, preserving both the amplitude and timing of trust changes, whereas the LDM and LSM tended to attenuate oscillations and produced overly smoothed trajectories that failed to capture trust fluctuations. On the other hand, all models generally captured the overall direction of change for the Drifting case; however, the NDM more accurately reflected the rate of trust evolution and intermediate deviations, while baseline models often lagged behind the self-reported criterion or underestimated gradual shifts. In cases of Stable dynamics, differences between models were less evident, with all approaches yielding relatively stable predictions, although the NDM maintained closer alignment with subtle variations in trust over time.

These examples illustrate that the advantage of the NDM becomes most evident when trust exhibits meaningful temporal structure, whereas the baseline models are sufficient only when trust remains nearly stationary.

**Figure 7:**
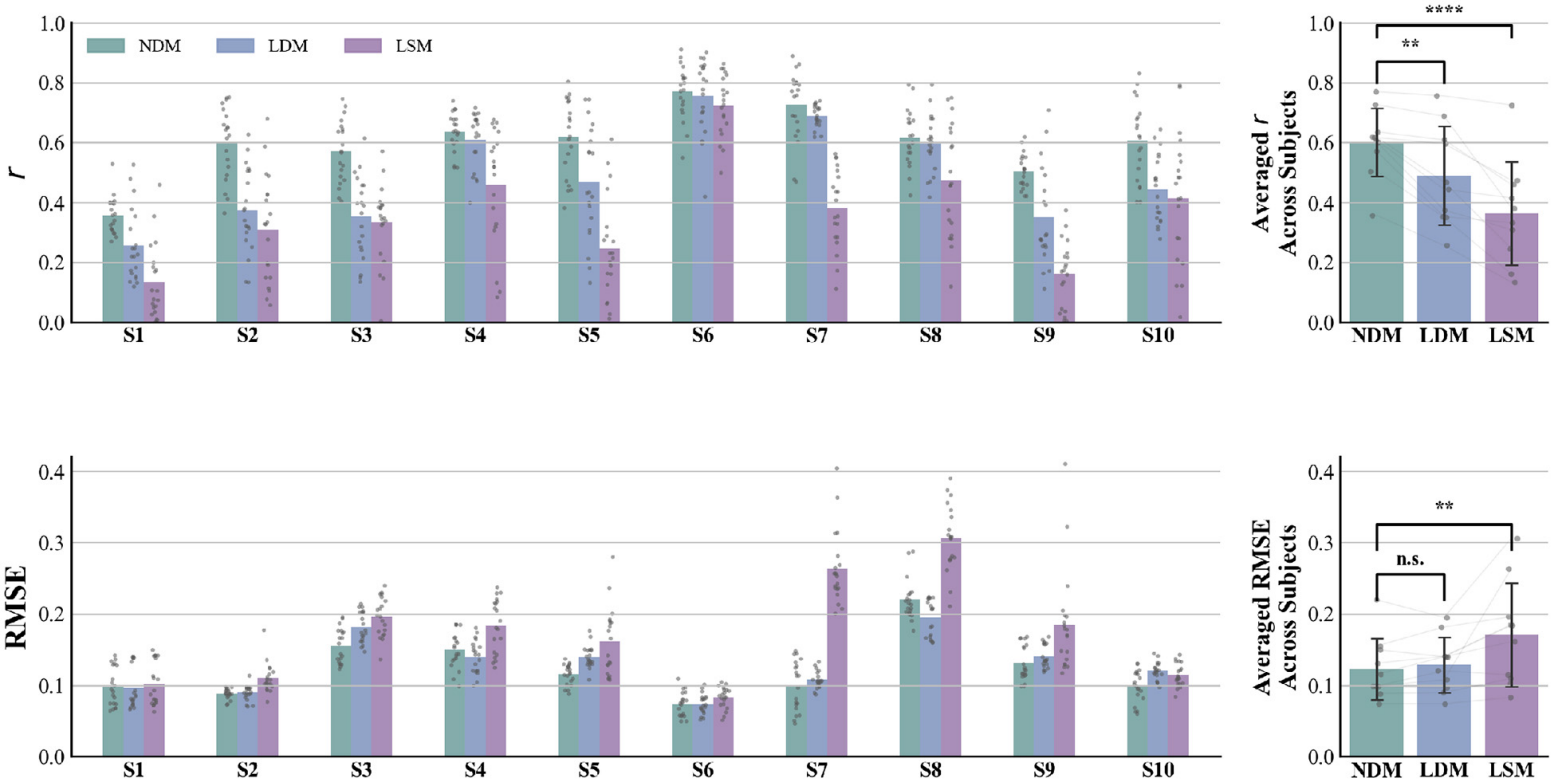
Model performance comparison across subjects (individual, **left**; averaged, **right**). Prediction accuracy was evaluated using Pearson's correlation coefficient (*r*, top) and root mean square error (RMSE, bottom) for each subject (S1–S10). **Left-panel** bars show each subject's mean performance across cross-validation iterations, with iteration-level values shown as descriptive scatter points. **Right-panel** bars show means across the ten subject-level averages, with individual subject averages overlaid. Statistical significance between models was computed on subject-level averages only (^*^p < 0.05, ^**^p < 0.01, ^***^p < 0.001, ^****^p < 0.0001; n.s., not significant).

Further, the model performance was compared in detail on both individual and group levels. Figure 7 summarizes the prediction performance of the three models which are evaluated using Pearson correlation coefficient (*r*) and root mean squared error (RMSE) across all subjects. Across subjects, the NDM consistently achieved higher correlation coefficients compared to both baseline models. While the magnitude of improvement varied across individuals, the NDM showed a clear advantage in capturing the temporal structure of trust signals, particularly for subjects exhibiting more pronounced temporal variability. To avoid pseudoreplication from repeated cross-validation folds, group-level tests were conducted on subject-level averages. In contrast, LDM and LSM generally produced lower correlation values and showed reduced sensitivity to temporal changes in trust. A complementary pattern was observed for RMSE. The NDM yielded lower prediction error for most subjects, whereas LSM exhibited the largest errors overall. Although some subjects showed comparable RMSE between NDM and LDM, the participant-level RMSE difference between these two dynamic models was not statistically significant.

The averaged results further highlight these trends. When averaged across subjects, the NDM significantly outperformed both LDM and LSM in terms of higher *r*. For RMSE, the NDM significantly reduced prediction error relative to the static baseline, while its RMSE reduction relative to the LDM was numerically favorable but not statistically significant. These findings suggest that incorporating non-linear neural representation learning into a dynamic modeling framework provides measurable benefits, especially for tracking the temporal structure of trust.

The results of statistical comparisons are presented in Table 2. Normality of pairwise subject-level performance differences was first assessed using the Shapiro-Wilk test. For *r*, the subject-level differences were consistent with normality, and paired *t*-tests revealed statistically significant improvements of the NDM over both baseline models. Specifically, NDM achieved higher correlation than LDM (NDM: *M* = 0.600; LDM: *M* = 0.489; *t*(9) = 4.26, *p* = 0.0021) and LSM (LSM: *M* = 0.364; *t*(9) = 7.25, *p* < 0.0001). A Fisher *z*-transformed sensitivity analysis led to the same conclusion. For RMSE, NDM had the lowest numerical error (NDM: *M* = 0.123; LDM: *M* = 0.128; LSM: *M* = 0.170). The RMSE reduction relative to LSM was significant (Wilcoxon *W* = 0, *p* = 0.0020), whereas the RMSE difference between NDM and LDM was not significant (*t*(9) = −1.11, *p* = 0.297). Thus, after correcting the unit of analysis, the NDM showed robust improvement in temporal correlation and reduced error relative to the static baseline, while the RMSE advantage over the linear dynamic baseline should be interpreted as descriptive rather than statistically reliable.

**Table 2:** Participant-level statistical comparison of trust prediction performance between models.

Model comparison	Correlation (*r*)					RMSE			
	Test	Test statistic	Mean Diff.	95% CI	*p*-Value	Test	Test statistic	Mean Diff.	*p*-Value
NDM vs. LDM	paired *t*-test	*t*(9) = 4.26	0.111	[0.052, 0.170]	0.0021	paired *t*-test	*t*(9) = −1.11	−0.0056	0.297
NDM vs. LSM	paired *t*-test	*t*(9) = 7.25	0.237	[0.163, 0.311]	< 0.0001	Wilcoxon	*W* = 0	−0.0476	0.0020

### 4.4 Model interpretation

**Figure 8:**
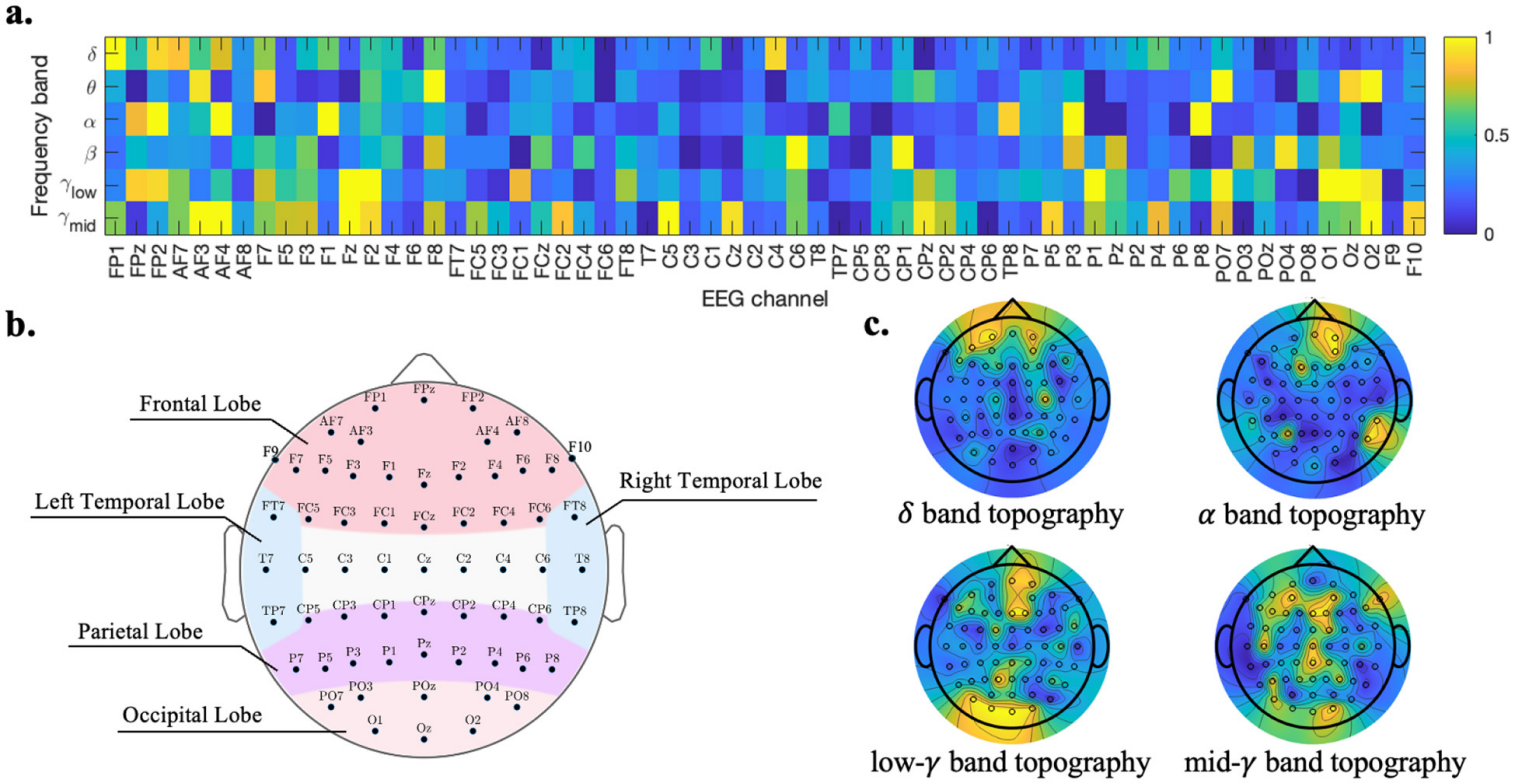
EEG feature importance across frequency bands and EEG channels. **(a)** Heatmap of normalized feature importance averaged across subjects and trials, with rows representing frequency bands (δ, θ, α, β, low-γ, mid-γ) and columns representing EEG channels. **(b)** 10–20 EEG channel locations with brain region annotations. **(c)** Representative topographic maps illustrating the spatial distribution of averaged feature importance for selected frequency bands (δ, α, low-γ, mid-γ).

To further examine the neural correlates of trust in human-autonomy teaming, feature importance was analyzed to characterize the contributions of different EEG spectral features in this section. Figure 8 presents the EEG feature importance estimated using LIME and visualized as a channel-frequency heatmap. Importance values were averaged across subjects and normalized to facilitate comparison. The resulting map demonstrates a clearly non-uniform distribution of feature relevance across both spatial and spectral dimensions, indicating that the model selectively weighted specific EEG components rather than relying uniformly on all channels or frequency bands.

From a spectral perspective, feature importance differed not only in magnitude but also in spatial distribution across frequency bands. Delta and alpha band features exhibited more spatially coherent and consistent importance patterns, particularly over frontal and fronto-central regions. In contrast, gamma-band features (low-γ and mid-γ) displayed numerous high-importance values but with greater spatial heterogeneity, characterized by localized, channel-specific contributions rather than broad regional patterns.

Spatially, the most highlighted features were concentrated over frontal and prefrontal regions, with a pronounced emphasis along the midline electrodes such as FPz, Fz, and FCz. Additional contributions were observed in central and parietal areas, whereas occipital channels generally showed reduced importance across most frequency bands. This spatial pattern indicates a dominant role of anterior cortical regions in supporting trust inference.

To further clarify the spatial organization of the most informative spectral components, Figure 8c shows scalp topographies for selected frequency bands. The delta-band topography exhibits a clear frontal and fronto-central dominance, reinforcing the significance of anterior low-frequency activity. The alpha-band topography displays a more distributed pattern extending from frontal into parietal regions, while gamma-band topographies are comparatively heterogeneous and spatially diffuse.

All in all, these findings suggest that the model primarily leverages slow cortical dynamics in frontal and fronto-central regions to infer trust, with higher-frequency activity providing supplementary, spatially localized information. This combination of dominant low-frequency frontal contributions and secondary gamma-band effects highlights a multiscale neural signature underlying trust dynamics in human-autonomy interaction.

**Figure 9:**
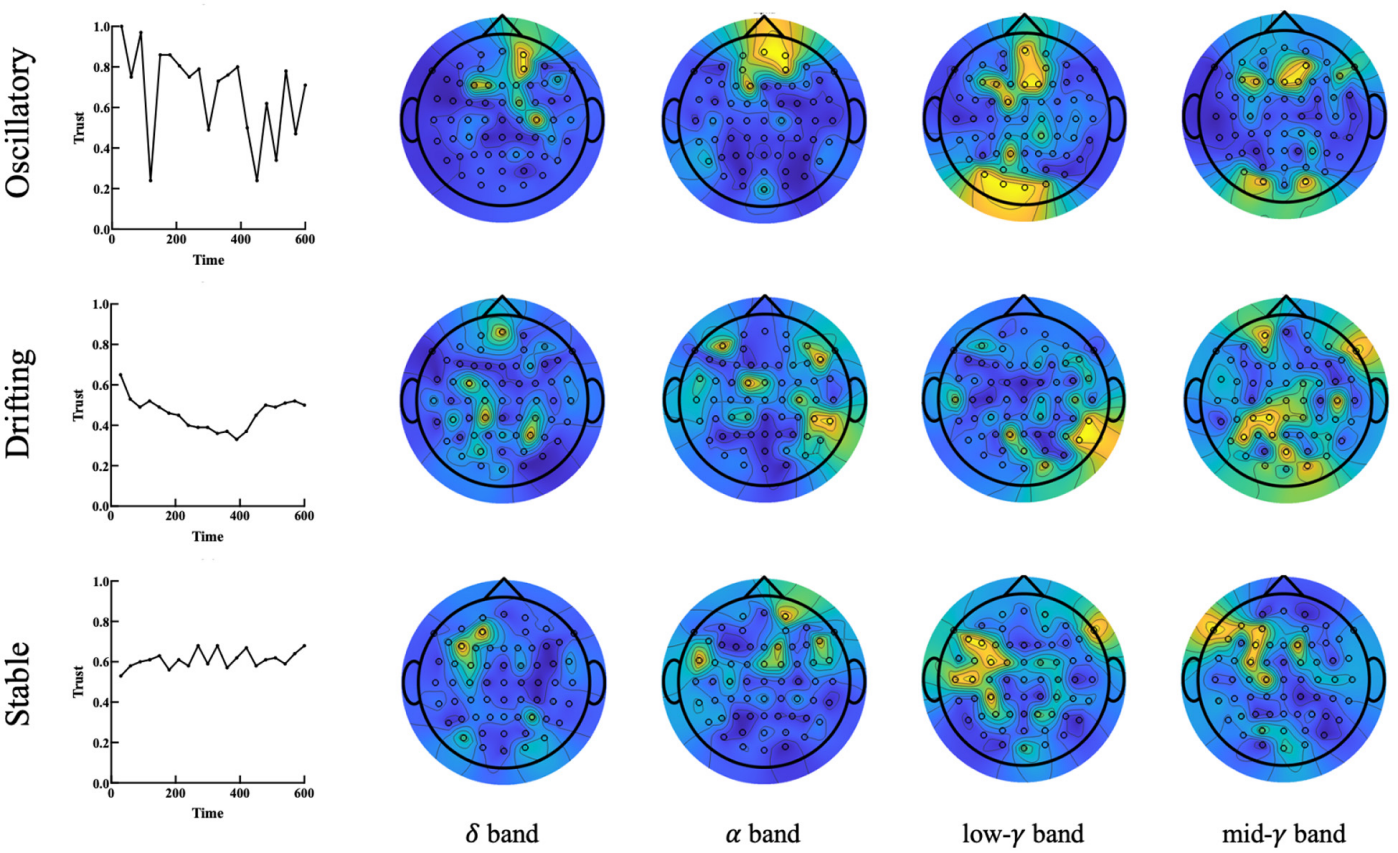
Representative EEG feature importance patterns associated with oscillatory, drifting, and stable trust dynamics. Rows correspond to three characteristic trust dynamics illustrated by representative trust trajectories (**left**). For each dynamic type, band-specific EEG topographies (δ, α, low-γ, mid-γ) show the spatial distribution of feature importance on the scalp topographies.

To examine whether neural feature contributions vary with different trust temporal dynamics, three representative trials were selected corresponding to the previously identified *Oscillatory, Drifting, and Stable* trust profiles (Figure 9, left column). For each profile, scalp topographies are shown for selected frequency bands that exhibited prominent contributions in the group-level analysis, enabling a direct comparison between trust dynamics and associated neural patterns. Across all three trust profiles, a common feature emerges in the form of consistent frontal and prefrontal delta-band importance. This shared spatial pattern suggests that slow frontal neural activity constitutes a core component of trust inference that is largely preserved across individuals, regardless of how trust evolves over time. Such consistency indicates that delta-band features may encode a subject-independent baseline process related to monitoring, expectation, or internal state maintenance during human-autonomy interaction.

Beyond this shared low-frequency features, distinct spatial-spectral distributions are observed across trust dynamic types. For the Oscillatory trust profile, feature importance extends more strongly into higher-frequency bands, particularly low- and mid-gamma activity over frontal and parietal regions. These localized gamma-band contributions may reflect high sensitivity to rapid changes in system behavior or frequent reassessment during unstable trust dynamics. In contrast, the Drifting profile exhibits a broader distribution of importance across alpha and low-gamma bands, with involvement extending toward parietal and temporal regions, consistent with gradual trust updating and accumulated integration of task feedback over time. For the Stable one, feature importance is characterized by broadly distributed low-frequency activity, particularly in delta and alpha bands, alongside more localized contributions from higher-frequency bands. This pattern is consistent with a conserved internal trust state supported by stable low-frequency dynamics, with some salient but localized involvement of higher-frequency activity.

Together, these results suggest that the model captures a shared low-frequency neural correlate for trust inference, while incorporating additional, localized high-frequency features in a dynamics-dependent manner to accommodate individual differences in trust behavior. This adaptive weighting of neural features highlights the importance of modeling both common and subject-specific components when inferring trust in dynamic human-autonomy teaming context.

**Figure 10:**
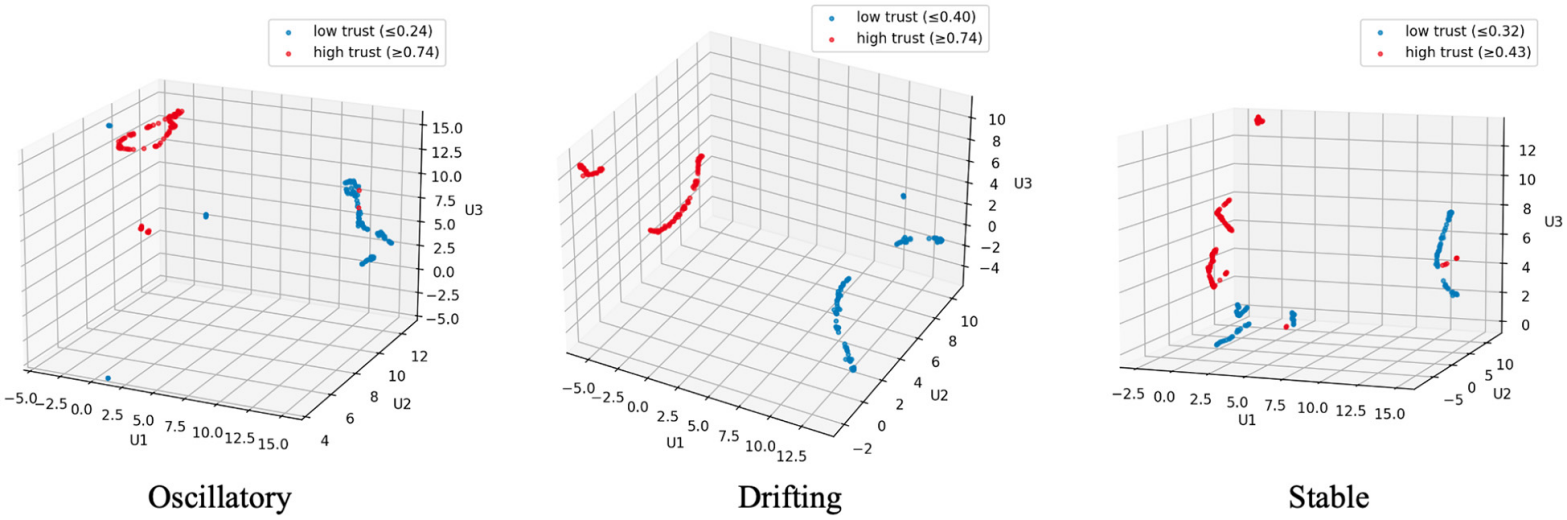
Supervised UMAP applied to the smoothed latent state *x*_*smooth*_ inferred by the proposed model for representative Oscillatory, Drifting, and Stable trust profiles. Points are colored by high-trust (red) and low-trust (blue) states, defined using subject-specific percentile thresholds.

To further examine how trust-related information is encoded in the inferred latent dynamics, supervised UMAP was applied to the smoothed latent state trajectories for three representative trust dynamic types as well. Across all three trust dynamics, high- and low-trust samples formed distinct regions within the embedded latent space, indicating that the inferred latent state captures trust-related structure beyond simple temporal smoothing. However, the geometric organization of these regions differed systematically across trust types in Figure 10. For Oscillatory trust dynamics, high- and low-trust states formed compact and well-separated clusters, suggesting rapid transitions between distinct latent regimes corresponding to abrupt trust changes. In Drifting trust dynamics, the latent embedding exhibited smoothly varying structures, with high- and low-trust samples distributed along continuous trajectories, consistent with gradual trust evolution. The latent states in Stable trust dynamics remained within a constrained subspace, with high- and low-trust samples separated by relatively short patterns, reflecting limited trust variability over time. These results indicate that the learned latent state captures both trust level and temporal structure, while preserving differences in how trust evolves across individuals.

## 5 Discussion

### 5.1 EEG-based trust predictive models

This study evaluated three EEG-based trust inference models with increasing levels of structural complexity: a non-linear dynamic model (NDM), a linear dynamic model (LDM), and a linear static model (LSM). After correcting the unit of statistical analysis to the participant level, the primary performance advantage of the NDM was most robust for temporal correlation with self-reported trust. The NDM significantly improved correlation relative to both the LDM and LSM, indicating that the non-linear dynamic model better captured the time-varying structure of trust reports than either baseline. RMSE results were more conservative: NDM significantly reduced error relative to the static model, but the RMSE difference relative to the linear dynamic model did not reach significance. These results suggest that incorporating both non-linear neural representations and temporal dynamics provides measurable advantages for inferring trust during HAT. The performance gap between the NDM and LDM highlights the importance of non-linear representation learning when working with high-dimensional EEG features. While the LDM accounts for temporal evolution via a latent state-space structure, its strictly linear observation model appears insufficient to capture the complex mappings between neural activity and trust. In contrast, the manifold learning component of the NDM enables a more expressive encoding of EEG features before temporal filtering, leading to improved predictive performance. Comparisons with the LSM further underscore the necessity of dynamic modeling. Despite benefiting from sparsity via LASSO regularization, the static regression approach lacks leveraging temporal continuity in trust evolution. This limitation is particularly evident for subjects exhibiting oscillatory or drifting trust dynamics, where trust values depend strongly on prior states rather than instantaneous neural features alone. Therefore, these findings support the view from previous research that trust is not a purely reactive or instantaneous construct, but instead evolves as a temporally structured internal state (Lee and See, 2004; Xu and Dudek, 2015).

Importantly, performance improvements were not uniform across subjects. Some individuals showed relatively small differences between models, particularly those with stable trust trajectories. This observation reinforces the role of individual variability in trust formation and suggests that the benefits of dynamic modeling are most pronounced for participants whose trust is more adaptive or volatile over time.

### 5.2 Model interpretation and trust-related neural correlations

Beyond predictive performance, feature-importance analyses provide insight into the neural correlates of trust captured by the proposed model in this study. The importance heatmaps revealed that trust inference relied on distributed EEG features spanning multiple frequency bands and cortical regions, rather than being dominated by a small subset of channels. This distributed pattern aligns with prior findings that trust engages both cognitive and affective processes (Lee and See, 2004; Dimoka et al., 2012), supported by networks involving frontal, temporal, and parietal regions.

Band-specific topographies further indicated that lower-frequency activity, particularly in the delta and alpha bands, contributed substantially to trust prediction, with pronounced involvement of frontal and prefrontal electrodes. These frequencies have been associated with attentional engagement, mental workload, and affective regulation (Klimesch, 1999; Cavanagh and Frank, 2014; Borghini et al., 2014), all of which are relevant to trust appraisal during interaction with autonomous systems. At the same time, contributions from low- and mid-gamma bands were also observed, suggesting that higher-frequency activity associated with information integration, cognitive control, and decision-making processes may play a complementary role in updating trust (Jensen et al., 2007; Fries, 2015).

When examined in the context of representative trust dynamics, distinct neural patterns emerged. Subjects exhibiting Oscillatory trust dynamics tended to show more spatially dispersed feature importance, potentially reflecting high sensitivity to moment-to-moment system behavior (Xu and Dudek, 2015). In contrast, Drifting trust trajectories were associated with more consistent band-specific patterns, aligning with gradual belief updating and evidence accumulation over time (Lewicki et al., 2006). Stable trust dynamics corresponded to relatively uniform and lower-variance feature importance, consistent with conserved internal trust states.

In addition to feature-level interpretation, the geometric structure of the inferred latent dynamics was examined using supervised manifold visualization. Supervised UMAP embeddings of the smoothed latent state revealed clear separation between high- and low-trust samples across all three representative trust dynamics, indicating that trust-related information is explicitly organized within the learned latent space. Notably, the geometry of this separation differed across trust profiles: Oscillatory trust dynamics exhibited compact and well-separated latent regions consistent with rapid switching between trust states, Drifting trust dynamics showed smoother latent structures suggestive of gradual trust evolution, and Stable trust dynamics occupied more constrained subspaces reflecting limited variability. These findings provide complementary evidence that the proposed model captures trust as a structured, time-evolving internal state, with latent representations that preserve both trust level and temporal organization.

While these interpretations remain exploratory, they illustrate how model-based feature attribution can bridge predictive modeling and neurocognitive interpretation. Rather than identifying a single “trust center” in the brain, the results support a network-level view of trust, where multiple frequency bands and regions jointly contribute to trust evaluation and updating over time.

### 5.3 Limitations and future work

Several limitations of this study should be acknowledged. First, continuous slider reports provide an operational self-report criterion for trust, but they are not an objective ground truth for the latent cognitive state. Although standardized instructions and repeated within-subject measurements were used to improve interpretability, future work should combine continuous self-report with behavioral reliance, intervention decisions, and post-trial trust questionnaires. Second, the sample included 10 participants in an intensive multi-session EEG design. This design provided rich within-participant temporal data but limited population-level generalizability. Accordingly, group-level statistics were revised to use participant-level averages, and the results should be replicated in a larger independent cohort. Third, although the model used a low-dimensional latent representation, small MLPs, constrained hyperparameter grids, and cross-validation, overfitting remains a concern for flexible non-linear models trained on modest EEG datasets. Feature importance was obtained using *post hoc* attribution applied to model outputs. While this approach enables interpretation of the learned neural representations, it does not establish causal relationships between EEG activity and trust. Moreover, averaging feature importance across subjects may attenuate individual-specific effects, particularly in light of the heterogeneous trust dynamics observed across participants.

In addition, the present analysis focused on spectral power features derived from single-channel EEG signals. Other neural representations, such as functional connectivity, cross-frequency interactions, or network-level measures, were not considered. These features may capture complementary aspects of neural processing related to trust and could further enhance both predictive performance and interpretability in future work.

Finally, the experimental task involved a specific operationally inspired human-autonomy teaming scenario with simulated autonomous systems. Although this setting provides ecological relevance, the extent to which the findings generalize to other tasks, interaction modalities, or autonomy levels remains an open question. Future studies could evaluate the proposed modeling framework across a broader range of human-autonomy contexts and explore adaptive or personalized extensions that explicitly account for individual differences in trust dynamics.

## 6 Conclusion

This study examined the feasibility of inferring human trust in autonomous systems from EEG signals during an operationally motivated human-autonomy teaming task. By integrating neural feature extraction, dynamic modeling, and interpretable analysis, the results demonstrate that trust can be characterized as a temporally evolving cognitive state with identifiable neural signatures.

A non-linear dynamic modeling framework was introduced to capture the temporal structure of trust from high-dimensional EEG features. Compared with linear dynamic and static baseline models, the proposed approach achieved significantly higher participant-level correlation with self-reported trust. It also significantly reduced RMSE relative to the static model, while the RMSE reduction relative to the linear dynamic model was not statistically reliable after participant-level aggregation. These findings support the view that trust is not a purely reactive or instantaneous construct, but rather an internal state shaped by prior experience and ongoing interaction.

Analysis of trust distributions and temporal trajectories revealed substantial individual variability, with representative Oscillatory, Drifting, and Stable trust dynamics observed across participants. Feature-importance analyses further indicated that trust inference relies on distributed neural activity across multiple frequency bands and cortical regions, with prominent contributions from frontal and prefrontal areas. What's more, the latent-space visualization using supervised manifold learning showed that high- and low-trust states occupy distinct regions of the inferred latent space, with geometry that varies systematically across trust dynamics. Together, these results point to a network-level neural basis of trust that reflects the interaction of cognitive, affective, and attentional processes.

Overall, this work advances EEG-based trust inference by demonstrating the value of dynamic, interpretable modeling for capturing both common and individual-specific aspects of trust. By combining temporal modeling, feature-level attribution, and latent-state visualization, the proposed framework provides a foundation for trust-aware autonomous systems that can monitor and adapt to human trust states in real time, supporting more effective and resilient human-autonomy teaming.

## Data Availability

The raw data supporting the conclusions of this article will be made available by the authors, without undue reservation.
